# Chemical Properties, Fatty-Acid Composition, and Antioxidant Activity of Goji Berry (*Lycium barbarum* L. and *Lycium chinense* Mill.) Fruits

**DOI:** 10.3390/antiox8030060

**Published:** 2019-03-10

**Authors:** Prodromos Skenderidis, Dimitrios Lampakis, Ioannis Giavasis, Stefanos Leontopoulos, Konstantinos Petrotos, Christos Hadjichristodoulou, Andreas Tsakalof

**Affiliations:** 1 Department of Medicine, Lab of Hygiene and Epidemiology, University of Thessaly, Papakyriazi 22, 41222 Larissa, Greece; pskenderidis@uth.gr (P.S.); xhatzi@med.uth.gr (C.H.); atsakal@med.uth.gr (A.T.); 2 Department of Biosystems Engineering/Agricultural Technology, Technological Educational Institute of Thessaly, 41110 Larissa, Greece; aar99sl@teilar.gr (S.L.); petrotos@teilar.gr (K.P.); 3 Department of Food Technology, Technological Educational Institute of Thessaly, 43100 Karditsa, Greece; igiavasis@teilar.gr

**Keywords:** goji berry, total carbohydrate content, carbohydrate content, phenolic content, fatty acids

## Abstract

In this study, the content composition and antioxidant activity of goji berry fruits from two species (*Lycium barbarum* and *Lycium chinense*) were assessed. The total carbohydrate and phenolic contents were evaluated using attenuated total reflection Fourier-transform infrared (ATR-FT-IR) spectroscopy, while the antioxidant activity of fruits was examined with two in vitro methods, which are based on the scavenging activity of the 2,2-diphenyl-1-picrylhydrazyl (DPPH•) and 2,2’-azino-bis(3-ethyl-benzthiazoline-sulfonic acid) (ABTS•^+^) free radicals. The fatty-acid profile was determined using gas chromatography coupled with mass spectrometry (GC-MS). The results of this study indicate that the fruits of *L. barbarum* present higher concentrations in carbohydrates and phenolics than *L. chinense* Mill. fruits. Furthermore, the antioxidant activity based on the half maximal inhibitory concentration (IC_50_) measurements of DPPH• and ABTS•^+^ free-radical scavenging was higher in *L. barbarum* than *L. chinense* Mill. Also, the GCMS analysis confirms the high levels of linoleic, palmitic, and oleic acids contained in the fruits of both species. Finally, the results of this study clearly show that the concentration of bioactive and antioxidant molecules is higher in *L. barbarum* than in *L. chinense* fruits, which was also confirmed by ATR-FT-IR measurements.

## 1. Introduction

Plants and their fruits contain a wide variety of biological active secondary metabolites and, consequently, they are used for the development of drugs, dietary supplements, and functional foods, because of their antioxidant, anti-inflammatory, and antimicrobial properties [1,2]. *Lycium barbarum* is a perennial deciduous shrub with ellipsoid orange-red berries and a sweet-tangy flavor, also known as goji berry, wolfberry, barbary wolfberry, and Chinese boxthorn (or gouqizi in Chinese). The *Lycium* genus includes up to 70 species that vegetate in regions from the temperate to the subtropical regions of Eurasia, Australia, southern Africa, and North and South America [3]. *Lycium* species have a long history in China as they were used as medicine and functional food, and they are also referred to in the Traditional Chinese Pharmacopeia (TCP). Among the functional natural components, Goji berry fruits contain *L. barbarum* polysaccharides (LPB) that are water-soluble glycoconjugates, and they are the most well-researched components [4]. In addition to LBP, Goji berry fruits also contain carotenoids, wherein zeaxanthin dipalmitate is the predominant component (55.44% of total carotenoids), as well as polyphenols, which include caffeic acid, chlorogenic acid, *p*-coumaric acid, quercetin, and kaempferol [5,6,7,8].

It should be noted that extensive research was carried out on the bioactivity of *L. barbarum*, while only a few papers are published for the *L. chinense* fruits [9,10,11,12]. Moreover, there are no reported studies on fatty acids (FA) for fruits of Greek origin. Free FA and complexes of lipids have key roles in metabolism as essential components of all membranes, as gene modulators, and as important energy sources, since they produce large quantities of ATP, carrying chemical energy within cells for metabolism [13]. Τhe geographical origin is a significant quality parameter for many plant foods, since variations in soil composition, climate, and cultivation techniques cause differences in a plant’s chemical composition. The protected designation of origin (PDO) of some foods is of great importance in order to add high value to the product [14]. 

Currently, *L. barbarum* fruit is consumed by people in many ways, including drinking juice, eating raw and/or smoothies, mixed with tea, and added to trail mix, cereals, muffins, energy bars, or soups, and not only in China, where more than 95,000 tons of goji fruits are produced and derived from 82,000 hectares [15]. In recent years, the goji berry cultivation expanded to other countries, such as Greece, due to the rising demands of consumers for superfoods and goji berry-related food products.

Regarding the growing interest in introducing goji cultivation in different areas in Greece, the sustainability of its production compared with the imported fruit should be evaluated. This study emphasizes the chemical fingerprint composition of the two varieties of fruits; however, it is also necessary to consider nutraceutical features, defining effective extraction processes in order to produce highly bioactive extracts that can be used in the pharmaceutical and food industry. For this reason, the aim of current study was to determine the physicochemical properties, such as the total carbohydrate and phenol content, present in goji berry fruit varieties (*L. barbarum* and *L. chinensis* Mill.) collected from the Thessaly region in central Greece, compared to imported fruit, measured and evaluated based on attenuated total reflection Fourier-transform infrared (ATR-FT-IR) spectra. In addition, the fatty-acid composition was estimated by gas chromatography coupled with mass spectrometry (GC-MS) analysis, while two in vitro methods (2,2-diphenyl-1-picrylhydrazyl (DPPH) and 2,2’-azino-bis(3-ethyl-benzthiazoline-sulfonic acid) (ABTS)) were used for the evaluation of antioxidant activity. To the best of our knowledge, this is the first work that presents the concentration of the non-polar lipids of Greek goji berries obtained from the Thessaly region, and their chemical properties.

## 2. Materials and Methods

### 2.1. Goji Berry Samples

Goji berry fruits were collected from plantations of *L. barbarum* and *L. chinense* varieties located in the region of Thessaly in Greece. In total, eight samples were analyzed, six from the Thessaly region of Greece, one *L. barbarum* sample imported from Mongolia and another (*L. barbarum*) from China. More specifically, the three Thessaly samples of *L. barbarum* variety were collected during the months of June (LbC_1_), August (LbC_2_), and October (LbC_3_), while the other three Thessaly samples (LcC_1_, LcC_2_, and LcC_3_) were collected on the same days from the *L. chinense* plantation. All Thessaly samples were dried in order to reach a final moisture value on the order of 14%, which was similar to that of the imported ones. All samples were prepared in powder form with the use of a hummer mill. 

### 2.2. Chemicals

In this study, methanol (MeOH), anhydrous sodium sulfate (Na_2_SO_4_), sodium chloride (NaCl), potassium hydroxide (KOH), methyl-*tert*-butyl ether (MTBE), and deionized water (>18 MΩ∙cm resistivity) were used. The aforementioned products were purchased from Sigma-Aldrich (Life Science Chemilab S.A., Athens, Greece). Supelco™ (Munich, Germany) 37 component fatty-acid methyl ester (FAME) mix was also used. The FAME mix contains the methyl esters of 37 fatty acids (catalog no. 47885-U) in a concentration of about 2% and 4% of each fatty-acid methyl ester. Palmitic acid methyl ester and tridecanoid acid in FAME mix were also purchased from Sigma-Aldrich in 6% and ≥99% concentrations, respectively.

Also, Folin–Ciocalteu, sodium carbonate in anhydrous crystal form, gallic acid, ethanol, 2,2-diphenyl-1-picrylhydrazyl (DPPH), methanol, and 2,2’-azino-bis(3-ethyl-benzthiazoline-sulfonic acid) (ABTS) were used as compounds and for antioxidant activity determinations.

### 2.3. Determination of Total Carbohydrate Content (TCC) of the Extracts

The phenol/sulfuric method according to Dubois et al. [16] was used for the determination of TCC. Briefly, 1 mL of each sample solution was mixed with 0.5 mL of phenol (4%) and 2.5 mL of sulfuric acid (95%); after an incubation of 10 min, the optical density was measured at 490 nm using a Thermo Scientific (Evolution 201) spectrophotometer (Thermo Scientific, Madison, WI, USA). The TCC was calculated on the basis of the calibration curve of d-glucose and expressed as g of carbohydrates/L of the extract. The linearity range of standard d-glucose was determined as 0.01 to 0.1 mg/L (*R^2^* = 0.9918). The equation was obtained by linear fitting of *y* = 8.0357*x* + 0.0111 (data not shown), where *y* is the absorbance at 490 nm and *x* is the concentration of d-glucose. The TCC was expressed as mg of d-glucose equivalent per g of dried extract (mg/g dry weight). 

### 2.4. Determination of Total Phenol Content (TPC) of the Extracts

Samples were aqueous ultrasound extracted according to the methodology of Skenderidis et al. [17]. The TPC of the extracts was determined using the Folin–Ciocalteu microscale protocol [18]. Briefly, 20 μL of goji berry extract was added to 1.58 mL of deionized water and 100 μL of Folin–Ciocalteu reagent. After the immediate addition of 300 µL of Na_2_CO_3_ solution (20% *w*/*v*), the mixture was left for 120 min of incubation in the dark. Finally, the optical density was measured at 765 nm using the abovementioned spectrophotometer. TPC was expressed as mg of Gallic Acid Equivalent (GAE) per g of dried fruit weight (mg GAΕ/g dry weight) after determination using a standard curve of absorbance values derived from standard concentration solutions of GA.

### 2.5. Determination of Total Antioxidant Capacity of Goji Berry Fruit

#### 2.5.1. DPPH (2,2-diphenyl-1-picrylhydrazyl) Radical-Scavenging Activity

The DPPH radical-scavenging activity of the samples was evaluated according to the method described by Skenderidis et al. [17]. Dissolved in distilled water at different concentrations, goji berry samples were mixed with 1 mL of a freshly made methanol solution of DPPH• radical (100 μM). The contents were vigorously mixed and incubated at room temperature in the dark for 20 min and the absorbance was read at 517 nm. Methanol solutions of tested extracts and DPPH• were used as blank and control measurements, respectively. All experiments were carried out three times on two separate occasions. The percentage of radical-scavenging capacity (RSC) of the tested extracts was calculated according to the following equation:

% DPPH• radical scavenging activity = ((Abs*_control_* − Abs*_sample_*)/Abs*_control_*) × 100

where Abs*_control_* and Abs*_sample_* are the absorbance values of the control and the tested sample, respectively. The half maximal inhibitory concentration (IC_50_) value was calculated using the graph-plotted RSC percentage against extract concentration in order to compare radical-scavenging efficiency of the extracts. All experiments were repeated three times on two separate occasions.

#### 2.5.2. ABTS•^+^ (2,2’-Azino-bis-(3-ethyl-benzthiazoline-sulfonic Acid) Radical-Scavenging Activity

ABTS•^+^ radical-scavenging activity was measured according to the method described by Kerasioti et al. [19]. The reaction was carried out in 1 mL of final volume, containing 400 μL of H_2_O, 500 μL of ABTS (1 mM), 50 μL of H_2_O_2_ (30 μM), and 50 μL of 6 μΜ solution of the enzyme horseradish peroxidase (HRP). Contents immediately after enzyme addition were thoroughly mixed and incubated for 45 min in the dark at room temperature. After 45 min of incubation in the dark, different concentrations of the tested sample solution were added and mixed thoroughly; then, the absorbance was measured at 730 nm. In each experiment, blank (samples without HRP) and control samples (without goji berry extract) were used. All experiments were repeated three times and on at least two separate occasions. Furthermore, the RSC percentage of the ABTS•^+^ radical-scavenging activity and IC_50_ values were determined as described above. 

### 2.6. Chemical Components and Structure Analysis by Infrared Spectroscopy

The FT-IR measurements were obtained at room temperature using a Nicolet 6700 (Thermo Scientific) spectrophotometer in order to estimate the chemical composition of the samples. The OMNIC 9 software (Thermo Scientific) was used for the configuration of the spectrophotometer equipped with an attenuated total reflectance (ATR) accessory, a deuterated triglycine sulfate (DTGS) detector (Thermo Scientific), and a KBr beam splitter (Thermo Scientific). A diamond crystal sampling plate Smart iTX, (Thermo Scientific) clamped with a pointed tip was used in order to place and analyze the samples. Background (empty sample plate) scans were acquired and rationed against the sample spectrum. Furthermore, the ATR crystal and pointed tip were cleaned in order to remove any interference from the preceding sample. Spectra were collected at 4 cm^−1^ spectral resolution in the mid-infrared range of 4000–650 cm^−1^ with 32 successive scans. Furthermore, all spectra were ATR- and baseline-corrected, normalized, and transformed to absorbance spectra. 

### 2.7. Fatty-Acid Methyl Ester (FAME) Synthesis

The method of FAME synthesis was applied as described by Gerasopoulos et al. [13]. Firstly, 0.5 mL of sample was placed in heat-resistant pyrex glass (16 cm length, 1.6 cm diameter) sealed with a teflon screw cap. As an internal standard, 1 mL of methanolic solution of tridecanoidacid (C13:0) was added at a concentration of 600 μg/mL. Subsequently, 0.4 mL of 10 N KOH (potassium hydroxide) and 2.7 mL of pure methanol were added. The examined solution was contained in tubes placed in a water bath at 55 °C for 90 min, while vigorous stirring every 20 min followed in order to achieve proper hydrolysis of the samples. Tap water was used in order to cool down the tubes for about 15 min. Fatty-acid methyl esters, 0.3 mL of 24 N H_2_SO_4_ were added in order to correct the composition. Thereafter, tubes were placed again in a water bath at 55 °C for 90 min followed by vigorous stirring every 20 min. A cooling bath with tap water followed, as previously. Finally, 3 mL of hexane was added as a solvent, and the samples were stirred with a vortex for 3 min. The treated samples were then placed in the centrifuge at 6000× *g* for 15 min at room temperature, and the supernatant solution (hexane layer containing the FAME) was placed in GC vials of 2 mL. Finally, the supernatant solution was stored at −20 °C until GS/mass spectrometry (MS) analysis was carried out.

#### GC-MS Analysis

The fatty-acid profile assessment in goji berry samples was carried out in duplicate. A standard solution containing 37 FAMEs was used to identify the individual FA supplied by Supelco^TM^ (Sigma-Aldrich, Munich, Germany) known as the 37 Component FAME Mix Standard. The peak area of the samples that was corrected with the respective correction factors was used for the calculation of the percentage of each FA [20]. The GC-MS Agilent 7890A GC chromatography apparatus (Agilent, Frankfurt, Germany) was used. The GC silica column (J&W 112-88A7: 804.11246 HP-88 250 °C: 100 m × 250 µm × 0.25 µm) (Agilent, Frankfurt, Germany) was located inside a temperature-controlled oven (Agilent, Frankfurt, Germany). The following MS parameters were used: interface temperature, 250 °C; MS ionization mode; helium gas with 45.2 mL/min flow; electron ionization; detector voltage acquisition mass range, 20–850 amu. Data were collected and processed using the GCMS Agilent integrated software (Agilent).

### 2.8. Statistical Analysis

The standard deviation was calculated and the averaged values along with the standard deviations (SD) are documented in the respective tables or figures. Statistical differences among the means, as well as interactions between the variables used in chemical analyses, were detected by one-way Analysis Of Variance (ANOVA) followed by Tukey’s test, and the statistical significance was set at *p* ≤ 0.05. MiniTab^®^17.1.0 software (Minitab LCC, State College, PA, USA) was used as the tool to perform the abovementioned tests.

## 3. Results and Discussion

Since *L. barbarum* fruits of different areas showed different contents of the main active components, their main medicinal and functional use should be consequently different [21]. 

The current study was conducted to investigate the chemical profiles of two varieties of the genus *Lycium* cultivated in central Greece by measuring the TCC and TPC, fatty-acid profile, and the antioxidant activity based on the ability to scavenge the free radicals of DPPH• and ABTS•^+^. 

### 3.1. Total Carbohydrate and Phenol Content

The TCC and TPC of the samples are presented in Table 1. According to the results, *L. chinensis* sample had lower TCC than *L. barbarum*, with values of 395 ± 4.1, 440 ± 5.2, and 329 ± 2.7, and 452 ± 3.8, 490 ± 6.8, and 370 ± 4.3 (mgGlu/g dry fruit), respectively, for each specie per collection month. The results for the imported fruits from China and Mongolia were 459 ± 3.8 and 434 ± 4.3, respectively. This is in line with the literature confirming that *L. barbarum* fruits have higher TCC [22].

The comparison of all samples shows that *L. barbarum* fruits that were collected in August show higher TCC, while the Mongolian sample had the lowest TCC. The highest rate of TCC of the Thessaly fruits confirms a previous study of our laboratory in which a significantly higher percentage of polysaccharides was present in *L. barbarum* [14].

Concerning TPC, *L. barbarum* the results were 9.7 ± 0.2, 10.1 ± 0.3 and 6.9 ± 0.3 compared to *L. chinense* with 8.5 ± 0.4, 8.9 ± 0.7, and 7.4 ± 0.6 mg GAΕ/g. Furthermore, the values of the imported from China fruits was 9.9 ± 0.6, while, for the Mongolian fruit, the value was 10.9 ± 0.3 mg GAE/g. The values of total polyphenol content obtained in the present study was higher than the values of 2.59–4.14 mg GAΕ/g presented by Ionică et al. [23], lower than the values of 106.80 ± 0.46 mg GAΕ/g reported from Kosar et al. [24], but well correlated with the values of 8.95–10.36 mg GAΕ/g for goji berries that were reported in the study of Medina [25]. 

### 3.2. Antioxidant Activity Based on DPPH• and ABTS•^+^

DPPH• and ABTS•^+^ are assays that measure the sample’s ability in deactivating radical species through electron transfer reactions [13,26,27]. The free-radical-scavenging activities determined by DPPH• and ABTS•^+^ are expressed as the IC_50_ value which is the effective concentration of the samples required to inhibit 50% of the initial free radical. Results are shown in Table 1. 

The DPPH• free-radical-scavenging capacities ranged from 784 to 1254 μg/mL, while the ABTS•^+^ values ranged from 192 to 407 μg/mL. As observed from the results, both *Lycium* species displayed good antioxidant activity. The best IC_50_ values of *L. chinense* and *L. barbarum* were achieved in August and, more specifically, they were 950 ± 4.7 and 830 ± 5.4 μg/mL for the DPPH•, while, for ABTS•^+^, the values were 220 ± 6.1 and 195 ± 3.5, respectively. The fruits imported from Mongolia showed a lower statistical IC_50_ value for DPPH•, while, for ABTS•^+^, they also showed a lower but not significant level when compared with Thessally *L. barbarum* fruits. The reported IC_50_ of DPPH radical-scavenging activity values are lower compared to the 42.76 ± 0.25 mg/mL reported by Benchennouf et al. [8] for their water fraction extract. 

Data presented in Table 1 showed that the antioxidant capacity of dehydrated *L. barbarum* fruits was higher compared to that of the *L. chinense* fruits cultivated in Greece, and the values are well correlated with the results obtained for the total phenolic content. The Mongolian sample showed the highest antioxidant activity of all samples investigated in this work against DPPH scavenging. The reported values and this correlation are in accordance with previous studies [23,28,29].

### 3.3. Goji Berry Fatty-Acid Profile

The fatty-acid composition of the goji berry samples is presented in Table 2 and is expressed as a percentage (%) of the total fatty acids (TFA). Goji samples of *L. chinense* collected from Thessaly region during October (LbC_3_) were found to have the greatest percentage of saturated fatty acids (SFA) (31.26%), while, in the same period, collected fruits of *L. barbarum* (LbC_3_) had the lowest percentage 26.1%. From both *Lycium* species collected in Thessaly, linoleic acid (C18:2 n-6) was the dominant polyunsaturated fatty acid (PUFA), ranging from 36.96% of the LcC_3_ sample (*L. chinense* collected in Thessaly in October) to 43.96% of the LbC_3 sample_ (*L. barbarum* collected in Thessaly in October), while the Mongolian sample had the lowest percentage of palmitic acid among all the samples investigated. Furthermore, the abovementioned results are consistent with the results reported by Blasi et al. [30].

The content of monounsaturated fatty acids (MUFA) ranged from 18.61% of LbC_1_ to 26.19% of the Chinese origin sample_._ Oleic acid was found to be the major MUFA with a percentage ranging from 16.71% of LbC_1_ to 22.39% of LcC_3_. Similar resultswere also reported by Cossignani et al. [12], who analyzed Italian, Chinese, and Mongolian goji berry fruits, and by Endes et al. [10] for Turkish origin goji berry fruits.

Polyunsaturated fatty acids (PUFA) were the most represented FA ranging from 43.23% of the LcC_3_ sample to 51.11% of LbC_3_, and these results are in line with those reported by Blasi et al. [30]. The α-linolenic acid (n−3) PUFA ranged from 5.39% of the Mongolia origin *L. barbarum* sample to 8.85% of LbC_2_. 

The ratios of PUFA/SFA and n−6/n−3 are significant for human health status and, as recommended by the World Health Organization [31], the PUFA/SFA ratio should be above 0.4–0.5. In this work, these ratios for all samples studied were found to be higher than 0.5, since their values ranged from 1.38 of the LcC_3_ sample to 1.95 of LbC_3_. It was suggested that the balance between the intake of n-6 and n-3 PUFA is more important than the levels of intake of individual FAs, with regard to many metabolic functions in the human body [32]. In our study, the ratio of n−6/n−3 ranged from 4.48 in LbC2 to 7.82 in the Mongolia origin *L. barbarum* sample.

The current study is the first attempt to evaluate the fatty-acid composition contained in Greek goji berries. Similar studies were completed by Jabbar et al. [33] in Ningxia goji berry fruits and pollen, and by Blasi et al. [30], who studied 16 commercial samples of fruits in Italy. The results of both studies are consistent with the results presented in this work.

### 3.4. ATR-FT-IR Spectroscopy

The fact that bioactive compounds present different ATR-FT-IR spectra denotes that infrared spectroscopy as an identification technique is of great importance for qualitative analysis. Functional groups and the structure characterization of all infrared peaks appearing in the spectra are presented in Table 2.

**Figure 1:**
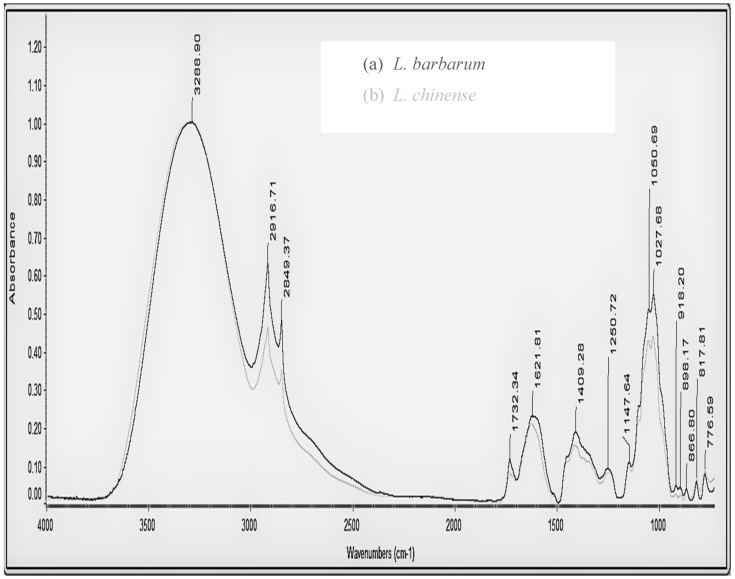
Typical attenuated total reflection Fourier-transform infrared (ATR-FT-IR) spectra of *Lycium barbarum* L. (a) and *L. chinense* Mill. (b) fruits cultivated in central Greece.

ATR-FT-IR spectra of LbC_2_ and LcC_2_ samples are presented in Figure 1. It can be said that there are no differences between the two fruits since both spectra are similar. The intensities of the peaks at ~1620 and 1732 cm^−1^ in the carbonyl region are correlated with the antioxidant activity (unpublished data), and this is in agreement with the literature data, where the powerful antioxidant nature of the compounds is explained by the fact that they possess a great number of compounds like organic acids and carbohydrates [34,35,36]. 

The identification of the major chemical groups of the examined compounds is usually based on the “fingerprint” region (950–1200 cm^−1^) of the spectra. The position (frequency) and intensity of the bands are characteristic of each compound, as presented in Table 3 [37]. The characteristic infrared band at ~898 cm^−1^ may be assigned to the β-anomeric configuration, while the bands at ~l028, 867, 818, and 777 cm^−1^ (Figure 1) can be attributed to the pyranose ring [38]. The higher intensities of the peaks that emerge in the spectrum of *L. barbarum* are probably related to the higher TCC and TPC content of this variety.

## 4. Conclusions

In the present study the composition of the bioactive substances of two *Lycium* species cultivated in Greece in different fruit periods of production were evaluated and compared with two samples which were imported from China and Mongolia. The results show that the Greek *L. barbarum* fruits from the Thessaly region contain higher amounts TCC and TPC than *L. chinense*. Among all samples, the Mongolian fruits showed better results against DPPH radical scavenging. Moreover, a significant variation in the fruit composition between the different cultivated periods emerged from the analysis results. The cultivated fruits in August showed higher amounts of the examined bioactive substances, which leads to the conclusion that this is probably due to the heat stress response of the plants because of the high temperatures during this particular month in the Thessaly area. Furthermore, the GC-MS analysis results show high levels of linoleic, palmitic, and oleic acids for the fruits of both species, which is in agreement with the results of other studies that appear in the literature. The analysis of the ATR-FT-IR spectra of the two varieties shows no differences between the two *Lycium* species. However, the intensity of the infrared peaks, which are related to the content of functional groups in the fruits, verifies the results obtained for the TP and TC contents. Nevertheless, for a better understanding of their phytochemicals and biological composition, as well as their effects, more investigations are needed.

## Figures and Tables

**Table 1:** Bioactive properties of *Lycium barbarum L*. and *L. chinense* Mill. fruits cultivated in central Greece and imported.

Parameters	*L. chinense* Mill.			*L. barbarum L.*			*L. barbarum L.*	*L. barbarum L.*
	June 2016 Collection	August 2016 Collection	October 2016 Collection	June 2016 Collection	August 2016 Collection	October 2016 Collection	Origin China	Origin Mongolia
TCC *	395 ± 4.1	440 ^a,b,c,d^ ± 5.2	329 ± 2.7	452 ± 3.8	490 ^a^ ± 6.8	370 ± 4.3	459 ^a,b,c,d^ ± 3.8	434 ^b,c,d^ ± 4.3
TPC *	8.5 ± 0.4	8.9 ^b^ ± 0.7	7.4 ± 0.6	9.7 ± 0.2	10.1 ^a,b^ ± 0.4	6.9 ± 0.3	9.9 ^a,b^ ± 0.6	10.9 ^a,b^ ± 0.4
IC_50_ of DPPH• *	1085 ± 2.9 μg/mL	950 ^a^ ± 4.7 μg/mL	1254 ± 5.1 μg/mL	894 ± 6.4 μg/mL	830 ^b,c,d^ ± 5.4 μg/mL	1150 ± 7.1 μg/mL	795 ^b,c,d,e^ ± 2.4 μg/mL	784 ^c,d,e^ ± 3.6 μg/mL
IC_50_ of ABTS•^+^ *	385 ± 2.9 μg/mL	220 ^a,b^ ± 6.1 μg/mL	407 ± 4.9 μg/mL	241 ± 7.7 μg/mL	195 ^a,b^ ± 3.5 μg/mL	397 ± 4.2 μg/mL	198 ^a,b^ ± 2.8 μg/mL	192 ^a,b^ ± 3.6 μg/mL

TCC: total carbohydrate content in mg/g dry weight (DW); TPC: total phenolic content in mg/g DW; * values are presented as means of ± standard deviation of at least three independent experiments; DPPH: 2,2-diphenyl-1-picrylhydrazyl; ABTS: 2,2’-azino-bis(3-ethyl-benzthiazoline-sulfonic acid); Half maximal inhibitory concentration (IC_50_) value showing the concentration that caused 50% scavenging of DPPH radical. Different letters indicate differences in the means within each raw estimate by Tukey pairwise comparisons. Means that do not share a letter are significantly different at *p* ≤ 0.05.

**Table 2:** Fatty-acid composition and nutritional ratios from goji berry samples.

Fatty Acids	Fatty Acids (%)	*L. barbarum L.*			*L. chinense* Mill.			*L. barbarum L.* Ningxia Origin	*L. barbarum L.* Mongolia Origin
		LbC_1_	LbC_2_	LbC_3_	LcC_1_	LcC_2_	LcC_3_		
Palmitic acid	C16:0	21.79 ± 0.0	21.55 ± 0.1	19.38 ± 0.6	21.59 ± 0.0	21.02 ± 0.2	24.67 ± 0.0	18.96 ± 0.0	15.08 ± 0.0
Palmitoleic acid	C16:1 n−9	1.90 ± 0.0	3.07 ± 0.0	1.66 ± 0.0	1.78 ± 0.0	1.51 ± 0.0	1.80 ± 0.1	1.01 ± 0.0	1.00 ± 0.0
Palmitoleic acid	C16:1 n−7	ND	1.80 ± 0.0	0.54 ± 0.1	0.55 ± 0.1	0.66 ± 0.1	0.58 ± 0.0	1.16 ± 0.1	1.17 ± 0.1
Stearic acid	C18:0	4.78 ± 0.2	4.05 ± 0.0	3.69 ± 0.1	3.43 ± 0.0	3.75 ± 0.0	4.20 ± 0.0	2.61 ± 0.0	2.69 ± 0.1
Oleic acid	C18:1 n−9	16.71 ± 0.1	17.32 ± 0.1	19.29 ± 0.0	20.08 ± 0.1	20.66 ± 0.1	22.39 ± 0.1	20.07 ± 0.1	19.61 ± 0.4
Linoleic acid	C18:2 n−6	42.64 ± 0.4	39.69 ± 0.3	43.96 ± 0.1	40.71 ± 0.0	38.65 ± 0.4	36.96 ± 0.1	37.89 ± 0.1	42.2 ± 0.1
Arachidic acid	C20:0	1.59 ± 0.0	1.03 ± 0.5	3.03 ± 0.0	1.14 ± 0.1	0.99 ± 0.0	1.15 ± 0.0	1.86 ± 0.0	2.03 ± 0.0
α-Linolenic acid	C18:3 n−3	7.99 ± 0.0	8.85 ± 0.3	7.15 ± 0.5	8.52 ± 0.0	7.47 ± 0.1	6.27 ± 0.0	6.46 ± 0.0	5.39 ± 0.2
Gondoic acid	C20:1 n−11	ND	1.41 ± 0.0	1.30 ± 0.1	0.89 ± 0.0	0.91 ± 0.2	0.74 ± 0.2	3.95 ± 0.2	4.05 ± 0.0
Behenic acid	C22	2.60 ± 0.0	ND	ND	0.56 ± 0.0	2.88 ± 0.2	0.48 ± 0.1	6.03 ± 0.0	6.78 ± 0.1
Lignoceric acid	C24	ND	1.23 ± 0.0	ND	0.75 ± 0.1	1.50 ± 0.1	0.76 ± 0.0	ND	ND
	ΣSFA	30.76	27.86	26.10	27.47	30.14	31.26	29.46	26.58
	ΣMUFA	18.61	23.60	22.79	23.30	23.74	25.51	26.19	25.83
	ΣPUFA	50.63	48.54	51.11	49.23	46.12	43.23	44.35	47.59
	PUFA/MUFA	2.72	2.05	2.24	2.11	1.94	1.69	1.69	1.84
	PUFA/SFA	1.64	1.74	1.95	1.79	1.53	1.38	1.50	1.79
	n6/n3	5.34	4.48	6.15	4.78	5.17	5.89	5.87	7.83

The reported values are mol.% mean values ± SD (*n* = 3), calculated using peak area values corrected with theoretical response factors. LbC_1_: *L. barbarum* collected in Thessaly in June 2016; LbC_2_: *L. barbarum* collected in Thessaly in August 2016; LbC_3_: *L. barbarum* collected in Thessaly in October 2016; LcC_1_: *L. chinense* collected in Thessaly in June 2016; LcC_2_: *L. chinense* collected in Thessaly in August 2016; LcC_3_: *L. chinense* collected in Thessaly in October 2016. SFA: saturated fatty acid; MUFA: monounsaturated fatty acid; PUFA: polyunsaturated fatty acid; ND: not detected.

**Table 3:** Peak analysis of the attenuated total reflection Fourier-transform infrared (ATR-FT-IR) spectra of the functional groups of *L. barbarum* L. and *L. chinense* Mill. fruits cultivated in central Greece.

*L. barbarum* Absorption (cm^−1^)	Peak Intensity	*L. chinense* Absorption (cm^−1^)	Peak Intensity	Functional Group	Structural Characteristic
3288.9	1	3296.7	1	hydroxyl group (–OH) amino group (–NH_2_)	stretching vibration of O–H stretching vibration of N–H
2916.71	0.631	2916.61	0.461	alkyl group (–CH_2_–)	stretching vibration of C–H
2849.37	0.480	2849.13	0.350	alkyl group (–CH_2_ or –CH_3_)	stretching vibration of CH_2_ and CH_3_
1732.34	0.115	1732.26	0.077	carboxyl group (–COOH), aldehyde group (–CHO) or esterfunction (–COOR)	stretching vibration of C=O
1621.81	0.229	1624.74	0.207	carbonyl group (–C=O or –CHO) amide group (–NH_2_ or –COR) amino group (–NH_2_)	stretching vibration of C=O bending vibration of N-H or stretching vibration of C=O bending vibration of N–H bound water
				alkyl group (–CH_2_- or –CH_3_)	bending vibration of C–H
1409.28	0.186	1416.25	0.151	carboxyl group (–COOH)	stretching vibration of C–O
1250.72	0.09	1245.96	0.08	carboxyl group (–COOH)	bending vibration of O–H
1147.64	0.107	1148.14	0.095	b-anomeric configuration	
1050.69 1027.68	0.51 0.55	1053.72 1030.17	0.426 0.438	hydroxyl group (–OH) D-glucopyranose ring	bending vibration of O–H symmetrical ring vibration
918.20	0.04	918.98	0.024	α-type glycosidic linkage	symmetrical ring vibration
898.17	0.03	898.12	0.022	b-anomeric configuration	
866.80	0.03	865.69	0.031	α-type glycosidic linkage	symmetrical ring vibration
817.81	0.05	817.64	0.063	α-D-galactopyranose	symmetrical ring vibration
776.59	0.07	776.99	0.08	D-glucopyranose ring	symmetrical ring vibration
